# Deciphering the Language of Intestinal Microbiota Associated with Sepsis, Organ Failure, and Mortality in Patients with Alcohol-Related Acute-on-Chronic Liver Failure (ACLF): A Pioneer Study in Latin America

**DOI:** 10.3390/microorganisms13051138

**Published:** 2025-05-15

**Authors:** Paula Alejandra Castaño-Jiménez, Tonatiuh Abimael Baltazar-Díaz, Luz Alicia González-Hernández, Roxana García-Salcido, Ksenia Klimov-Kravtchenko, Jaime F. Andrade-Villanueva, Kevin Javier Arellano-Arteaga, Mayra Paola Padilla-Sánchez, Susana Del Toro-Arreola, Miriam Ruth Bueno-Topete

**Affiliations:** 1 Departamento de Biología Molecular y Genómica, Instituto de Investigación en Enfermedades Crónico-Degenerativas, Centro Universitario de Ciencias de la Salud, Universidad de Guadalajara, Guadalajara 44350, Mexico; palejandra.2104@gmail.com (P.A.C.-J.); tonatiuhabd@gmail.com (T.A.B.-D.); ksenia.klimov@hotmail.com (K.K.-K.); mayrapadillasanchez@gmail.com (M.P.P.-S.); susana@cucs.udg.mx (S.D.T.-A.); 2 Unidad de VIH, Hospital Civil de Guadalajara “Fray Antonio Alcalde”, Guadalajara 44350, Mexico; luceroga08@gmail.com; 3 Departamento de Clínicas Médicas, Instituto de Investigación en Inmunodeficiencias y VIH, Centro Universitario de Ciencias de la Salud, Universidad de Guadalajara, Guadalajara 44350, Mexico; drjandradev@gmail.com; 4 Unidad de Urgencias Médicas, Hospital Civil de Guadalajara “Fray Antonio Alcalde”, Guadalajara 44350, Mexico; roxgarcia@hcg.gob.mx; 5 Unidad de Medicina Interna, Hospital Civil de Guadalajara “Juan I. Menchaca”, Guadalajara 44350, Mexico; javierarellano.md@gmail.com

**Keywords:** ACLF, microbiota, sepsis, organ failure, mortality, *Klebsiella/Faecalibacterium* ratio

## Abstract

ACLF is a severe stage of liver cirrhosis, characterized by multiple organ failure, systemic inflammation, and high short-term mortality. The intestinal microbiota (IM) influences its pathophysiology; however, there are currently no studies in Latin American populations. Therefore, we analyzed IM and its relationships with sepsis, organ failure, and mortality. In parallel, we quantified serum lipopolysaccharides as a marker of bacterial translocation. Fecal samples from 33 patients and 20 healthy controls (HCs) were obtained. The IMs were characterized by 16S-rRNA amplicon sequencing, the metagenomic functional predictive profiles were analyzed by PICRUSt2, and LPS quantification was performed by ELISA. Patients with ACLF showed significant alterations in alpha and beta diversity compared to the HCs. A strong dominance index accurately predicted 28-day and 90-day mortalities. The IMs showed a polarization toward Proteobacteria associated with increased LPS. The LPS correlated with clinical severity, organ dysfunction, and higher pathogenic taxa. The *Klebsiella/Faecalibacterium* ratio showed good performance in identifying sepsis (AUROC = 0.83). Furthermore, *Morganella*, *Proteus*, and *Klebsiella* were enriched in patients with multiorgan failure. *Lactobacillus*, *Escherichia*/*Shigella*, *Veillonella*, and *Ruminococcus gnavus* exhibited potential in predicting 28- and 90-day mortalities. The IM alterations in ACLF may be useful as clinical biomarkers of poor prognosis, primarily for mortality and sepsis. These findings are representative of western Mexico.

## 1. Introduction

Liver cirrhosis is the fourth-leading cause of death in Mexico, the main cause of which is alcohol misuse [1]. Approximately 30% of patients with cirrhosis may develop acute-on-chronic liver failure (ACLF), a syndrome characterized by acute decompensation of cirrhosis, multiple organ failure, and high short-term mortality. According to the CANONIC study [2], 28-day mortality in ACLF III patients can reach up to 73%, emphasizing the severity of this condition and the need for early and effective interventions.

Although the pathophysiological mechanisms of ACLF are not completely clear, it has been hypothesized that endogenous factors such as necroinflammation-derived molecules (DAMPs, damage-associated molecular patterns) and exogenous factors such as bacterial infections or PAMPs (pathogen-associated molecular patterns) play crucial roles in the progression of the disease [3].

In this context, our research group has previously shown that intestinal bacterial genera such as *Escherichia*/*Shigella* and *Prevotella* are significantly increased in patients with alcohol-related cirrhosis, whereas beneficial genera such as *Blautia* and *Faecalibacterium* are decreased [4]. However, to date, studies on IM in ACLF have focused mainly on American, European, and Chinese populations [5,6,7].

Intestinal microbiota dysbiosis has emerged as a central factor in the pathophysiology of liver diseases, as it promotes the disruption of the intestinal barrier and facilitates bacterial translocation [8,9,10]. This process allows for the passage of lipopolysaccharides (LPS), a potent endotoxin of Gram-negative bacteria, into systemic circulation, consequently activating exacerbated inflammatory responses [11,12].

In patients with ACLF, systemic inflammation is closely associated with the sepsis process, which accelerates organ dysfunction and worsens the prognosis, significantly increasing short-term mortality [11,13].

This clinical picture represents a significant challenge for medicine, since its timely diagnosis and adequate management remain limited, particularly in regions such as western Mexico, where the epidemiological characteristics and risk factors for ACLF in the population are still poorly studied. Furthermore, LPS has been postulated as a critical biomarker to evaluate the degree of inflammation and its clinical impact [12,14], although its specific role in the Mexican population with ACLF remains little explored.

The Mexican population has distinctive features that make it particularly interesting for this type of study. For example, the prevailing Western diet in Mexico is enriched in fats and carbohydrates [15,16], and its negative impact on inflammatory response is well known; on the other hand, genetic ancestry has shown that individuals with pre-Hispanic ancestry have a higher prevalence of ACLF, a higher mortality, and more severe outcomes compared to European–American and African–American populations with ACLF [17]. These important findings may be associated with a distinct intestinal microbiota compared to other populations, which could affect the inflammatory response and progression to ACLF.

Therefore, the present study aims to analyze the intestinal microbiota in patients with ACLF in western Mexico and explore its relationships with sepsis, organ failure, and short-term mortality. Furthermore, serum LPS levels were quantified as a potential biomarker of bacterial translocation and systemic inflammation. This approach seeks to provide novel evidence of the role of the intestinal microbiota in the pathophysiology of alcohol-related ACLF, contributing to the understanding of this serious syndrome in a vulnerable population.

## 2. Materials and Methods

### 2.1. Study Design and Recruiting Participants

This was a cross-sectional analytical study. Patient recruitment took place at the Civil Hospitals of Guadalajara, “Fray Antonio Alcalde”, and “Juan I. Menchaca” in Guadalajara, Jalisco, Mexico. It was conducted in accordance with the guidelines of the World Medical Association (Declaration of Helsinki, revised in 2013) and was approved by the Ethics and Research Committees of the aforementioned hospitals and the University Center for Health Science Committee (010/20, 00012, and 22–96, respectively). The purpose of the study was explained, and written informed consent was obtained from participants and/or their caregivers. Healthy participants were recruited from the same community.

The inclusion criteria for alcohol-related ACLF were as follows: (a) patients of both sexes diagnosed according to EASL clinical practice guidelines [18]; (b) aged between 18 and 70 years; and (c) body mass index between 18.5 and 29.9 kg/m^2^. The following were considered as exclusion criteria: the use of prebiotics/probiotics 4 weeks prior to enrollment; seropositivity to HIV, HVB, or HCV; hospitalizations within the last 3 months due to COVID-19; and gastrointestinal disorders or known autoimmune diseases, as well as oncological diseases.

The inclusion criteria for healthy subjects (HCs), as the control group, were as follows: (a) age between 18 and 70 years; (b) BMI between 18.5 and 29.9 kg/m^2^; (c) no current or past SARS-CoV-2 infection for at least 3 months prior to enrollment; (d) no use of prebiotics/probiotics 4 weeks prior to enrollment; (e) no use of antibiotics 3 months prior to enrollment; (f) no known allergies or intolerances to fiber sources; (g) non-vegetarians or -smokers; (h) alcohol consumption ≤28 g per week.

### 2.2. DNA Extraction and 16S rRNA Amplicon Sequencing

DNA was extracted from 150 mg of feces stored at −80 °C using the Quick-DNA Fecal/Soil Microbe Miniprep Kit (Zymo Research, Irvine, CA, USA) according to the manufacturer’s protocol. DNA was quantified with a NanoDropTM OneC spectrophotometer (Thermo Scientific, Waltham, MA, USA), and the purity, using the A260/A280 ratio, was also revised. The 16S rRNA amplicon-sequencing library’s preparation was performed according to the Illumina MiSeq protocol [19]. The V3 and V4 regions of the 16S rRNA gene were amplified by PCR following the manufacturer’s protocol with the Platinum Taq DNA Polymerase High Fidelity (Invitrogen, Waltham, MA, USA), as well as the following previously published primers: 341F: 5’TCGTCGGCAGCGTCAGATGTGTATAAGAGACAGCCTACGGGNGGCWGCAG-3’ and 805R: 5’GTCTCGTGGGCTCGGAGATGTGTATAAGAGACAGGACTACHVGGGTATCTAATCC-3’. The obtained amplicons were further purified with AMPure XP (Beckman Coulter, Indianapolis, IN, USA) magnetic beads and quantified with the Qubit 3 dsDNA HS kit (Invitrogen, Waltham, MA, USA) according to the manufacturer’s indications. Index incorporation was achieved with the Nextera XT Index Kit v2 Set A (Illumina, San Diego, CA, USA). Finally, indexed amplicons were pooled to equimolar concentrations into a 4 nmol/L solution tube. The resulting library was denatured and further sequenced using the MiSeq Reagent V3 600-cycle (Illumina, San Diego, CA, USA) according to the protocol.

### 2.3. Bioinformatic Analysis

Analyses of the 16S rRNA sequences were performed with the QIIME2 version 2024.2 amplicon distribution [20]. Sequences with Phred ≥30 (quality parameter) were accepted. Raw reads were further filtered by denoising with DADA2 via *q2-dada2* [21] using the default settings. Taxonomy assignation was performed, trained on our own sequences, using a naïve classifier (via *q2-feature-classifier*) [22], employing Silva 138.1 as a reference taxonomic database [23,24]. ASVs identified as mitochondria and chloroplasts were removed. Then, filtered ASVs were aligned using MAFFT via q2-alignment, and the phylogeny was built with FastTree2 via *q2-phylogeny* [25]. The alpha-diversity indices [26,27,28] and beta-diversity distances (unweighted and weighted Unifrac) [29,30] were computed via *q2-diversity*. PCoA plots were generated to visualize the beta-diversity distances using Emperor via *q2-emperor* [31] and further analyzed by PERMANOVA.

Differential abundance analyses at the genus level were accomplished by means of ANCOM-BC (via *q2-composition*), which is a compositional statistical method that accounts for the sampling fraction and normalizes read counts, while controlling for false discovery rates [32]. Before the analysis, a frequency filter was applied in which features that appeared more than 50 times in at least 10% of the samples were retained. A *q* ≤ 0.05 cutoff was used to assess the significance, as well as a log fold change (LFC) ≥ |1.0| to evaluate the effect size. To assess the potential metabolic profile of the intestinal microbiota, the PICRUSt2 pipeline [33,34,35,36,37] was employed, coupled with the MetaCyc Database [38]. The resulting pathways were further analyzed using ANCOM-BC, using the previously described parameters. The Proteobacteria/Firmicutes and *Klebsiella*/*Faecalibacterium* ratios were computed as previously published [39].

### 2.4. Statistical Analysis

Data normality was examined using the Shapiro–Wilk test. If significant, a parametric distribution was assumed and unpaired Student’s *t*-test or one-way ANOVA with a Bonferroni post hoc test was performed to assess the differences between the groups; if not, the Mann–Whitney U or Kruskal–Wallis with a Dunn’s post hoc test was performed. Beta-diversity distances were statistically analyzed by performing PERMANOVA tests. Both alpha- and beta-diversity statistical analyses were corrected with Benjamini–Hochberg (BH) multiple testing through the QIIME2 package. The Fisher’s exact test was applied to evaluate the categorical variables. We tested associations between clinical and microbiome continuous variables with Spearman rank correlation. Survival analysis for the selected microbiome parameters were performed using Kaplan–Meier curves, and the *p*-value was obtained from a log-rank test. Gehan–Breslow–Wilcoxon tests, hazard ratios, and median survival were also calculated. All statistical tests were two-tailed, and a *p*-value or false discovery rate-adjusted *q*-value (using the Benjamini–Hochberg test) equal to or less than 0.05 were considered statistically significant. Data were analyzed using SPSS 25.0. Plots were generated utilizing GraphPad Prism version 9.3.0.

### 2.5. LPS Quantification

Five microliters of blood was collected by venipuncture and then centrifuged at 1100× *g* for 10 min to obtain serum. Serum LPS levels were determined by immunoassays (ELISA) using the LPS (Lipopolysaccharides) ELISA Kit (FineTest, Wuhan, China, Catalogue no. EU3126) following the manufacturer’s recommendations. Dilutions of 1:5 for patients and 1:3 for controls were used.

## 3. Results

### 3.1. Cross-Sectional Study and Clinical Characteristics of Recruited Patients

A total of 53 participants were recruited from August 2022 to December 2023. These participants included 33 patients diagnosed with acute-on-chronic liver failure (ACLF) from the Emergency Department and Internal Medicine of the aforementioned hospitals. A total of 20 healthy controls (HCs) were also recruited (Table 1). No significant differences in age and BMI were observed between the different groups, confirming homogeneity in both study groups. As expected, significant differences were observed in the complete blood count parameters and in the markers of liver, kidney, and coagulation function between ACLF and HC patients. Markers of systemic inflammation, such as the platelet-to-neutrophil ratio, also showed significant differences between the two groups (*p* < 0.001).

All patients included in the study were classified as Child–Pugh C, with a median MELD-Na score of 33.5 (IQR 28–38). When stratifying patients based on ACLF grades, 72.7% were identified as severe ACLF, comprising grades 2 and 3. All patients were receiving treatment with lactulose or antibiotics. Hepatic encephalopathy (HE) stood out as the most frequent decompensation, observed in 84.8% of cases. Infections were present in 60.6% of patients, while sepsis affected 30.3%. Among organ failures, renal failure was the most frequent, while respiratory failure was the least common. Finally, a 28-day mortality rate of 39.4% was recorded.

### 3.2. Intestinal Microbiota Alpha and Beta Diversity

**Figure 1:**
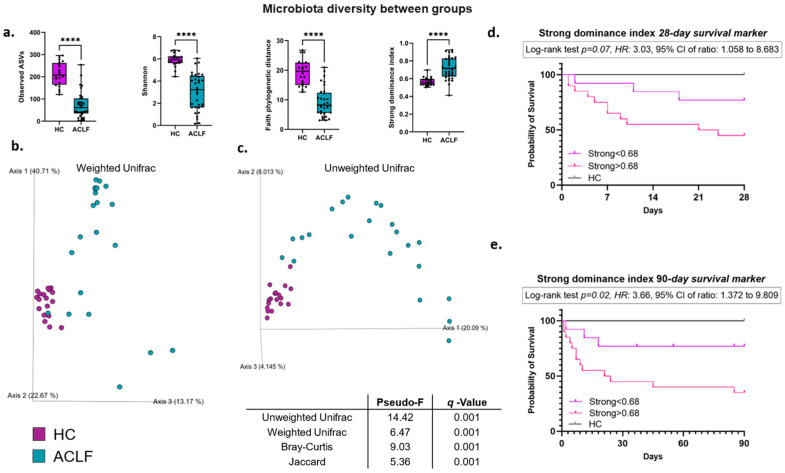
Alpha- and beta-diversity metrics of the intestinal microbiota and their association with mortality in ACLF patients. (**a**) Alpha-diversity indices: Observed ASVs, Shannon index, Faith phylogenetic distance, and Strong dominance index in HCs compared with ACLF patients. The boxes extend from the 25th to the 75th percentiles (interquartile range, IQR), and the lines inside the boxes represent the median values. Analyzed with the Mann–Whitney U test, **** *p* < 0.0001. (**b**,**c**) Principal coordinates plots (PCoAs) of the beta diversity, employing weighted and unweighted Unifrac distances. ACLF patients (blue dots); HCs (purple dots). Analyzed with PERMANOVA, *q* < 0.001 in both cases. (**d**,**e**) Kaplan–Meier analysis of 28- and 90-day survival of patients with ACLF, according to the Strong dominance index. A cutoff point of 0.68 was established using the median of the data, identifying the following two groups: patients with Strong < 0.68 (purple line) and Strong > 0.68 (pink line). The log-rank test (and its *p*-value), hazard ratio, and confidence intervals are shown for each case. The values for the control group (HCs) are included as references. Healthy controls (HCs); acute-on-chronic liver failure (ACLF).

The alpha-diversity metrics, which assess the richness, diversity, dominance, and phylogenetic diversity, show that the ACLF patients presented a significantly lower alpha diversity compared with the HCs (*p* < 0.0001, Figure 1a). Additionally, a highly relevant finding was that the progressive loss of alpha diversity in ACLF significantly impacted survival; this analysis was performed using Kaplan–Meier curves and the Strong dominance index, comparing survival at 28 and 90 days, establishing a cutoff point of 0.68 (Figure 1d,e). The Strong dominance index evaluates dominant taxa, showing higher values when the microbiota is polarized toward few bacterial species, correlating with a greater loss of equilibrium in the bacterial microenvironment.

This analysis reveals that at values below 0.68, the probability of survival is 77% at 28 and 90 days (Figure 1d,e, purple line). In contrast, when the Strong dominance index is greater than 0.68, the probability of mortality at 28 days is 45%, while at 90 days it drops to 35% (Figure 1d,e, pink line). Interestingly, the data show that 50% of the population dies within an average of 22.5 days, both at 28 and 90 days of follow-up. Furthermore, the Gehan–Breslow–Wilcoxon (GBW) test indicates that deaths of patients with Strong > 0.68 occur more rapidly during the early stages of follow-up, both at 28 and 90 days (*p* = 0.06 and *p* = 0.03). Finally, a Strong dominance index greater than 0.68 increases the risk of mortality in patients with ACLF by 3.03 and 3.66 times at 28 and 90 days, respectively. These data suggest a robust association between more pronounced dysbiosis and a clear increase in mortality. Also, the alpha-diversity analysis in stratified ACLF patients shows no significant differences (*p* = 0.36). Likewise, when analyzing clinical variables, such as hepatic encephalopathy, acute kidney injury (AKI), infections, or antibiotic use, among the ACLF group, no significant differences were identified between patients with and without these clinical features, only certain downward trends, using the Shannon index (Table S1).

Beta-diversity analyses were performed using weighted and unweighted Unifrac distances, which allow for quantitative and qualitative assessments of the phylogenetic differences between the studied groups (Figure 1b,c). The results are visualized using principal coordinates analyses (PCoAs), which show the clear formation of two well-defined clusters in both metrics. This suggests significantly distinct microbial profiles between the ACLF and HC groups, with statistically significant differences (PERMANOVA, *p* < 0.001). We additionally used other beta-diversity metrics such as Bray-Curtis and Jaccard, which confirmed these differences between groups (Figure 1, Table 1). Furthermore, different beta-diversity distances were analyzed in stratified ACLF patients, as well as the presence or absence of various clinical aspects, such as antibiotic use, hepatic encephalopathy, AKI, or infections. No significant differences (*q* > 0.1) were found according to the presence of these variables (Table S2), so the impact of these clinical variables on the ACLF patients is not significant.

### 3.3. Analysis of the Relative and Differential Bacterial Taxonomy of the Intestinal Microbiota

**Figure 2:**
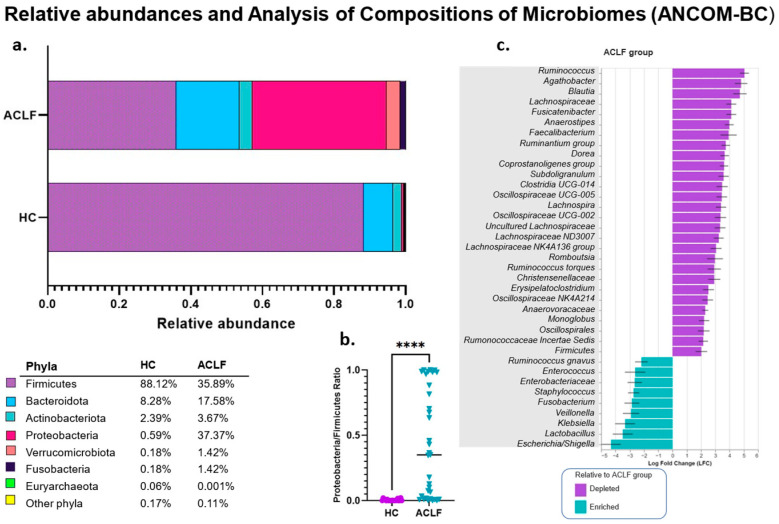
Bacterial taxonomy of intestinal microbiota in the different study groups. (**a**) Table and bar graphs represent the relative abundances at the phylum level in both study groups. (**b**) Scatterplot of the Proteobacteria/Firmicutes ratio, which were adjusted in such a way that a value close to 1 indicates a greater abundance of Proteobacteria, while a value close to 0 indicates a greater abundance of Firmicutes. The results were analyzed using the Mann–Whitney U test, **** *p* < 0.0001. (**c**) Differential analysis of the bacterial taxa of the ACLF patients and HCs at the family and genus levels by ANCOM-BC. The bars represent the log fold change (LFC) between both groups. Blue bars indicate the taxa of the ACLF group, while turquoise bars represent the HC group. Thresholds of |LFC| ≥ 2.0 and *q* < 0.05 were used. Healthy controls (HCs); acute-on-chronic liver failure (ACLF).

Bioinformatics analysis by relative abundances reveals significant shifts in bacterial populations at the phylum level between the ACLF and HCs (Figure 2a,b). The predominant phyla in the ACLF group were Proteobacteria (37.37% vs. 0.59% in HCs), Firmicutes (35.89% vs. 88.12%), Bacteroidetes (17.58% vs. 8.28%), Actinobacteria (3.67% vs. 2.39%), and Fusobacteria (1.42% vs. 0.18%).

Based on the observed taxonomic profile, the Proteobacteria/Firmicutes ratio was evaluated as a potential marker of dysbiosis in patients with ACLF. The results show a significant increase in this ratio in patients with ACLF compared to HCs, reinforcing its usefulness as an indicator of microbial alterations associated with this condition (*p* < 0.0001) (Figure 2b).

In addition, compositional analysis using the ANCOM-BC method was used to identify bacterial taxa that differentially characterize both study groups with robust statistical support. The results show that the representative taxa in the HC group mainly belong to the Firmicutes phylum, including genera such as *Clostridia*, *Blautia*, *Faecalibacterium*, *Agathobacter*, *Ruminococcus*, and *Fusicatenibacter*. In contrast, the ACLF group is characterized by a predominance of proinflammatory and pathogenic taxa from the Proteobacteria phylum, especially those from the Enterobacteriaceae family, such as *Escherichia/Shigella* and *Klebsiella*. In addition, other representative taxa were identified in these groups, such as the genera *Enterococcus*, *Lactobacillus*, *Veillonella*, *Fusobacterium*, and *Staphylococcus* (Figure 2c). These analyses are consistent with the relative abundances at the genus level (Figure S1).

### 3.4. Functional Metagenomic Profiles

**Figure 3:**
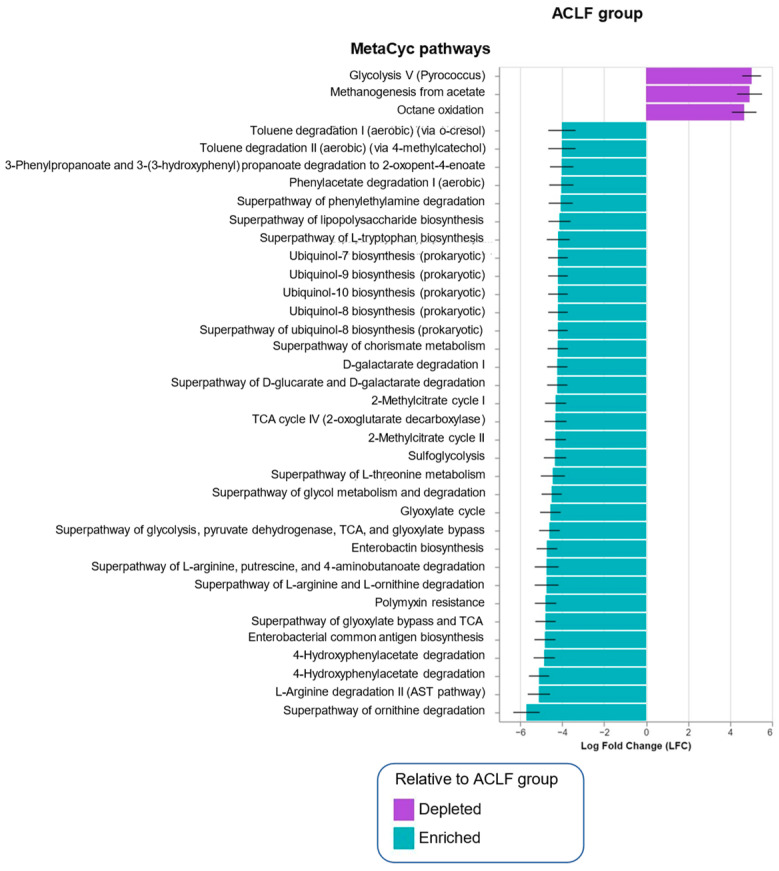
Functional predictive analysis of intestinal microbiota metabolic pathways in healthy controls (HCs) and ACLF patients assessed by PICRUSt2 and ANCOM-BC. Each bar represents a significantly enriched pathway in each group, consistent with the MetaCyc database. Purple bars represent pathways enriched in the HCs, and blue bars represent pathways enriched in the ACLF group. Thresholds of |LFC| ≥ 4 and *q* < 0.05 were used. Acute-on-chronic liver failure (ACLF).

The ANCOM-BC methodology was used to analyze the data generated by PICRUSt2 to predict the functional potential of bacterial communities based on the profiles obtained through marker gene sequencing, according to each study group. This analysis reveals a significant enrichment of specific metabolic pathways among the study groups (Figure 3). In healthy subjects (HCs), an increase in metabolic pathways related to glycolysis and octane oxidation was identified, while in patients with ACLF, a higher prevalence of pathways associated with the tricarboxylic acid cycle, antibiotic resistance (particularly polymyxins), and L-arginine degradation was observed.

Notably, patients with ACLF showed significant enrichment in pathways related to lipopolysaccharide biosynthesis, as shown in Figure 3. Additionally, enriched metabolic pathways were identified as being associated with the degradation of aromatic compounds, such as toluene, and with the biosynthesis of chorismate, which is an essential precursor for the bacterial synthesis of aromatic amino acids, such as L-tryptophan, L-phenylalanine, and L-tyrosine. These compounds are essential for important bacterial functions, including maintaining cell-wall integrity and the synthesis of kynurenine and kynurenic acid. Notably, these compounds can exert a neuroactive and proinflammatory effect on the host [40,41].

Finally, an increase was observed in pathways related to the metabolism of ubiquinones, molecules known for their role in the electron transport chain, as well as in the degradation pathway of 2-methylcitrate, a metabolite generated during the catabolism of amino acids and fatty acids.

### 3.5. Analysis of the Intestinal Microbiota Related to Sepsis, Organ Failure, and Mortality

**Figure 4:**
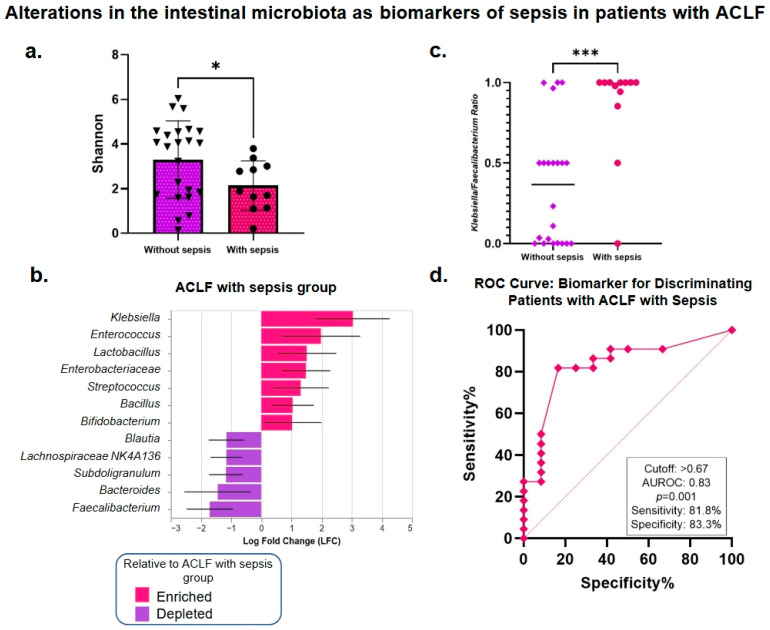
Alpha-diversity comparisons and differential analysis of the bacterial taxonomy of the intestinal microbiota in patients with sepsis-associated ACLF and its clinical utility. (**a**) Alpha diversity, as assessed by the Shannon index, associated with the presence or absence of sepsis. (**b**) Differential taxonomy analysis in the presence or absence of sepsis. Red bars show patients with sepsis, while purple bars represent patients without sepsis. (**c**) Scatter plot of the *Klebsiella/Faecalibacterium* ratio in patients with and without sepsis. This ratio indicates a greater abundance of *Klebsiella* when the value is closer to 1, while a value closer to 0 indicates a greater abundance of *Faecalibacterium*. The results were analyzed using the Mann–Whitney U test. (**d**) ROC curve showing the performance of the *Klebsiella/Faecalibacterium* ratio as a sepsis biomarker. The AUROC, sensitivity, and specificity values obtained in the analysis are presented. * *p* < 0.05, *** *p* < 0.001. Acute-on-chronic liver failure (ACLF).

One common complication that occurs in patients with cirrhosis is sepsis, a severe systemic inflammatory response to infection that can accelerate organ dysfunction and mortality in patients with ACLF. This analysis found that alpha diversity is significantly lower in patients with sepsis compared to those with ACLF without sepsis (*p* = 0.04) (Figure 4a). Interestingly, we observed that the bacterial taxa that predominate in the sepsis group are pathogenic, opportunistic, and aerobic, such as the Enterobacteriaceae family in general and some genera within it, such as *Klebsiella*, *Enterococcus*, *Streptococcus*, and *Bacillus*. In contrast, patients without sepsis retain some taxa with beneficial functions, such as *Faecalibacterium*, *Bacteroides*, and *Subdoligranulum* (Figure 4b). These findings suggest that altered intestinal microbiota composition could be a contributing factor to susceptibility to infections in patients with cirrhosis, specifically in the context of ACLF.

To identify a suitable biomarker for diagnosing sepsis in ACLF based on the microbiota profile, the *Klebsiella/Faecalibacterium* ratio was evaluated. These bacterial genera were selected based on the ANCOM-BC analysis’ findings as the most abundant and the least abundant significant genera (Figure 4b). Patients with sepsis were compared with patients without sepsis, showing a significant elevation in patients with sepsis (Figure 4c). Additionally, ROC curves were calculated to determine the potential of the *Klebsiella/Faecalibacterium* ratio as a biomarker for the diagnosis of sepsis (Figure 4d). The results show high performance in discriminating sepsis, with an area under the curve (AUROC) of 0.83. This model exhibited a sensitivity of 81.8%, indicating that most sepsis cases were correctly identified, and a specificity of 83.3%, reinforcing the biomarker’s accuracy in identifying sepsis in this population.

Simultaneously, subanalysis of the IM in relation to organ failure revealed no significant differences in bacterial richness and diversity, consistent with alpha-diversity metrics. However, beta-diversity distances showed significant differences among ACLF patients with different failures. Specifically, significant differences were observed in ACLF patients with and without coagulopathy using the weighted Unifrac distance (*p* = 0.05), as well as in patients with and without liver and circulatory failure, assessed using the Bray-Curtis distance (*p* = 0.03 and *p* = 0.05, respectively) (Figure S2, Table S3). Renal, brain, and respiratory failure did not exhibit changes in beta-diversity distances (Table S3).

**Figure 5:**
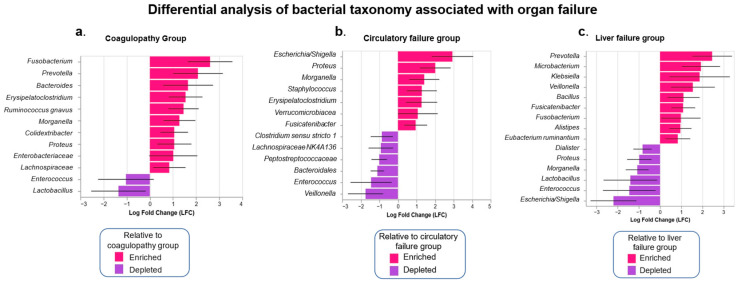
Differential analysis by ANCOM-BC of the bacterial taxonomy of the intestinal microbiota in ACLF, in the presence or absence of several organ failures: (**a**) coagulopathy; (**b**) circulatory failure; (**c**) liver failure. Presence of organ failure (red); absence of organ failure (purple). The bars represent the log fold change (LFC) between both groups. A threshold of |LFC| ≥ 0.8 was used. Acute-on-chronic liver failure (ACLF).

The ANCOM-BC analysis, which identifies statistically significant bacterial taxa, shows that the genera *Morganella*, *Proteus*, and *Erysipelatoclostridium* were enriched in patients with circulatory failure and coagulopathy (Figure 5a,b). Furthermore, some pathobionts enriched in patients with liver failure were *Prevotella*, *Klebsiella*, *Veillonella*, and *Fusobacterium* (Figure 5c). In contrast, *Enterococcus* and *Lactobacillus* were enriched in patients without any of these organ failures, whether liver, circulatory, or coagulopathy.

**Figure 6:**
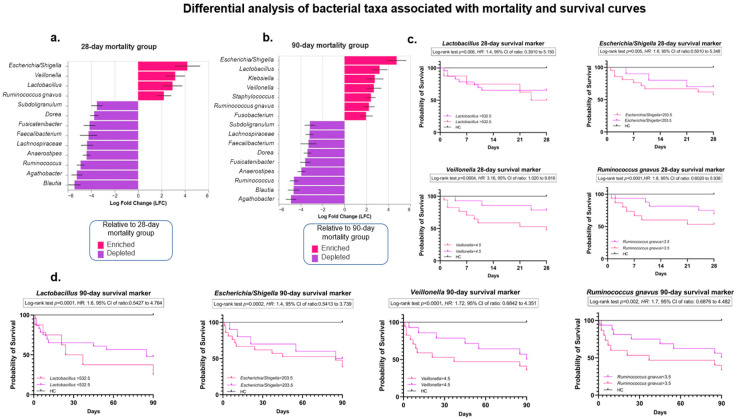
Differential taxonomy analysis of IM at the levels of bacterial families and genera associated with mortality and survival curves in patients with ACLF. Differential bacterial taxonomy of (**a**) 28-day mortality and (**b**) 90-day mortality of the ACLF group, compared to the HC group, analyzed by ANCOM-BC. The bars represent the log fold change (|LFC| > 2.0) between both groups. Red bars indicate taxa characteristic of the group that died, while purple bars represent depleted taxa (i.e., enriched in the HC group). (**c**,**d**) Kaplan–Meier mortality analyses of 28- and 90-day survival in patients with ACLF, according to the number of ASVs detected for Lactobacillus, Escherichia/Shigella, Veillonella, and Ruminococcus gnavus at 28 days (**c**) and 90 days (**d**). Data were transformed using CLR, and a cutoff point was subsequently established using the ROC curves for each taxon evaluated. The Log-rank tests (*p*) were calculated considering the HC curve. Hazard ratios and confidence intervals were calculated for the ACLF patients. Intestinal microbiota (IM); acute-on-chronic liver failure (ACLF).

Finally, to enhance these results, a differential taxonomy analysis was performed in ACLF patients who died at 28 and 90 days and compared with the HC group. Interestingly, the ANCOM-BC results show that the genera *Escherichia/Shigella*, *Veillonella*, *Lactobacillus*, and *Ruminococcus gnavus* were strongly associated with the patient groups that died at 28 and 90 days, compared with the HCs. In contrast, *Agathobacter*, *Ruminococcus*, *Blautia*, *Anaerostipes*, *Faecalibacterium*, *Fusicatenibacter*, and other taxa were depleted in the mortality group (and, therefore, enriched in the HC group) (Figure 6a,b).

On the other hand, the Kaplan–Meier mortality analysis revealed novel findings regarding 28- and 90-day mortality in ACLF patients, according to the presence or absence of mortality. The bacterial taxa determined by the ANCOM-BC analysis (*Lactobacillus*, *Escherichia/Shigella*, *Veillonella*, and *Ruminococcus gnavus*) and the number of ASVs associated with each taxon were considered.

Specifically, the ASV counts of *Lactobacillus* > 532.5, *Escherichia/Shigella* > 203.5, *Veillonella* > 4.5, and *Ruminococcus gnavus* > 3.5 were associated with a 28-day mortality probability of 50%, 57%, 47%, and 53%, respectively. Moreover, a Lactobacillus count of >532.5 ASVs suggests that 50% of the ACLF population has a median survival of approximately 26 days. Notably, *Veillonella* >4.5 ASVs increased the risk of 28-day mortality by 3.16 fold (CI 1.020 to 9.816).

As expected, the mortality rate in ACLF patients for all four taxa evaluated increased at 90 days, reaching 75% for *Lactobacillus*, 62% for *Escherichia/Shigella*, 65% for *Veillonella*, and 67% for *Ruminococcus gnavus*. At the initial stages of follow-up, a more accelerated mortality rate was observed in the presence of elevated ASVs counts of *Escherichia/Shigella*, *Veillonella*, and *Ruminococcus gnavus*, as shown in Figure 6c,d at both 28 and 90 days. These results have not been previously reported, even in the global literature, so the identification of *Veillonella*, *Ruminococcus gnavus*, *Lactobacillus*, and *Escherichia/Shigella* as potential biomarkers of poor prognosis highlights the importance of the intestinal microbiota in the pathophysiology of this disease.

### 3.6. Quantification of Serum LPS Concentrations

In patients with cirrhosis, alterations in the intestinal barrier and dysfunction of the immune system occur, favoring bacterial translocation and the release of its components, such as LPS, into the bloodstream. In the present study, serum LPS levels were quantified using ELISA. The results show that the average LPS levels were 3.79 ± 0.31 µg/mL in healthy controls (HCs) and 6.7 µg/mL ± 0.42 in patients with ACLF (*p* < 0.0001).

**Figure 7:**
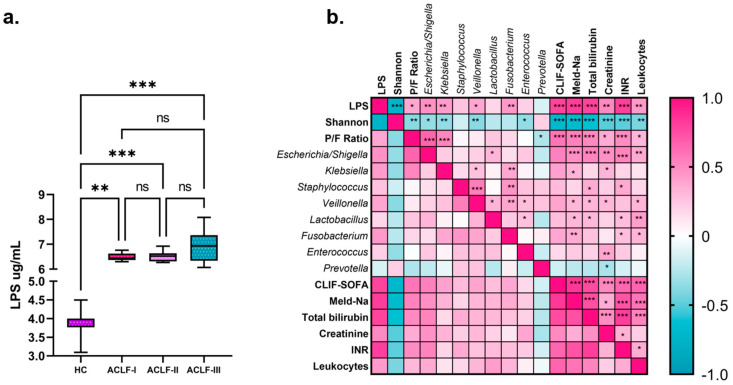
Serum LPS concentrations and correlation analysis among the study groups. (**a**) LPS concentrations in healthy controls (HC) and ACLF patients stratified into grades I, II, and III. Statistical analysis was performed using the Kruskal–Wallis test, with post hoc Dunn’s multiple comparisons test. (**b**) Correlation matrix assessing the relationship between LPS levels, intestinal dysbiosis markers, clinical severity indices, and parameters associated with organ dysfunction. Correlations were calculated using the Spearman test, using a color scale (pink, positive correlation; blue, negative correlation). Significant differences among groups: ns not significant, * *p* < 0.05, ** *p* < 0.01, *** *p* < 0.001. Acute-on-chronic liver failure (ACLF).

When the data were stratified according to the ACLF grade, it was found that patients with ACLF II showed a greater tendency toward elevation compared to patients with ACLF I and II (Figure 7a). These findings suggest that elevated LPS levels are closely related to disease severity, particularly in patients with more advanced ACLF.

Correlation analysis of the LPS levels with the representative taxa of the intestinal microbiota, clinical severity indices, biochemical markers, and leukocyte counts revealed several statistically significant correlations (Figure 7b). LPS levels were negatively correlated with alpha diversity (Shannon index) and positively correlated with the Proteobacteria/Firmicutes ratio, indicating pronounced dysbiosis. A positive correlation was also observed between the LPS levels and the MELD-Na and CLIF-SOFA indices, in addition to leukocytosis. These correlations also included biochemical markers such as creatinine, total bilirubin, and INR, which are indicators of renal and hepatic failures and coagulopathy, respectively. Furthermore, the LPS levels were positively associated with specific bacterial taxa, including *Escherichia/Shigella*, *Klebsiella*, *Veillonella*, and *Fusobacterium*, reinforcing their association with alterations in the intestinal microbiota, which is characteristic of patients with ACLF.

## 4. Discussion

Globally, alcohol consumption is one of the main factors responsible for cirrhosis-related deaths [42,43]. In Mexico, alcohol use disorder contributes to 32.8% of deaths associated with liver disease [1]. Cirrhosis is characterized by significant alterations in the gut–liver axis, particularly in advanced stages of the disease [44]. Our research group has pioneered the description of alterations in the intestinal microbiota in patients with alcohol-associated decompensated cirrhosis in western Mexico [4,45]. However, to date, dynamic changes in the intestinal microbiota in one of the most severe forms of cirrhosis, namely, ACLF, have not been investigated in this population. Therefore, the present study is representative and unique to our mestizo-Mexican population. It is important to highlight that studies on microbiota and ACLF also do not exist in Latin America. The present study describes potential alterations in the intestinal microbiota in patients with alcohol-related ACLF, emphasizing significant modifications related to sepsis, organ failure, and especially short-term mortality.

In the present study, the alpha diversity exhibited a highly significant decrease in ACLF patients compared to the healthy controls (HCs). Interestingly, we observed that the progressive loss of alpha diversity significantly impacted the survival rate. This important finding was demonstrated by the Strong dominance index and Kaplan–Meier curves, where values greater than 0.68 were associated with a 90-day survival rate as low as 35% in the ACLF patients, with accelerated mortality in the early stages of follow-up and a 3.03-fold increased risk of death in the ACLF patients. The Strong dominance index quantifies the dominance of one or more taxa relative to the entire community analyzed, with a value of one representing the maximum dominance concentration [46]. Consequently, higher values depict a polarized microbiota and indicate a profound inequality among bacterial communities, which, in this case, are associated with intestinal dysfunction. These findings suggest that a decreased alpha diversity is an important prognostic factor in ACLF. Regarding beta diversity, we observed evident changes in the taxonomic profiles with a significant phylogenetic distance between the ACLF group and HCs. These changes are consistent with the profiles previously described in the literature linking advanced cirrhosis and ACLF [4,6,7,44]. Regarding bacterial phyla, we observed an increased Proteobacteria/Firmicutes ratio in ACLF patients (Figure 2b), indicating the presence of an intestinal microbiota dominated by proinflammatory taxa. This index has been recently studied by our research group in the context of diseases such as obesity and HIV and correlates with microbiota profiles that have significantly lost bacterial groups associated with intestinal homeostasis, and it also emphasizes pathobiont/inflammatory groups [47,48]. Therefore, we suggest that this ratio could be useful as a marker of dysbiosis and endotoxemia in the current study population.

The differential taxonomy in our cohort, as analyzed by ANCOM-BC, demonstrated in the ACLF patients the characteristic hallmark of the Enterobacteriaceae family—such as *Escherichia/Shigella* and *Klebsiella*—and others, including *Enterococcus* and *Staphylococcus*, which have previously been linked to infectious and proinflammatory processes in patients with cirrhosis [49,50]. In contrast, beneficial genera such as *Faecalibacterium* and *Blautia* were significantly reduced in this group, which could reflect losses of essential protective functions of the microbiome such as bacterial fermentation of indigestible polysaccharides to produce short-chain fatty acids, such as butyrate. Butyrate, through the inhibition of histone deacetylase enzymes (HDACs), regulates cytokine expression in T cells and T-cell differentiation [51], as well as suppresses the expression of proinflammatory effectors in macrophages in the lamina propria [52]. In addition, it strengthens the intestinal barrier by inducing the expression of tight junction proteins, reducing intestinal permeability, and preventing bacterial translocation [53]. Additionally, butyrate acts as an energy source for colonocytes, promoting physiological hypoxia in the colon [52,54], an essential mechanism to limit the proliferation and translocation of bacteria, particularly Enterobacteriaceae.

On the other hand, *Escherichia/Shigella* and *Klebsiella* are the main pathogenic bacteria that produce LPS. This endotoxin is highly immunogenic, and its action is favored by the formation of biofilms. These biofilms increase the local concentration of LPS, promoting continuous release and protecting these bacteria so they can survive adverse environmental conditions and evade the immune system. Furthermore, these bacteria have fimbriae, structures that promote adhesion and intestinal translocation, especially in patients with cirrhosis [55,56,57,58,59]. These aspects are relevant since it has been shown that resistance to ceftazidime and biofilm production by *K. pneumoniae* are strongly associated with the biosynthesis of guanosine pentaphosphate (p)ppGpp [60]. Importantly, these metabolic pathways were significantly elevated in the ACLF group (Figure 3).

Bacterial translocation is considered a trigger of the characteristic systemic inflammation in these patients, which impacts the development of sepsis and organ failure, increasing short-term mortality [10,11,13]. Our results demonstrate a substantial increase in systemic LPS in patients with ACLF, which, combined with the predictive functional analysis of the intestinal microbiota using PICRUSt2, reinforces the importance of bacterial translocation as a trigger of systemic inflammation. Interestingly, we found a positive correlation between increased serum LPS levels and greater clinical severity, reflected through the MELD-Na and CLIF-SOFA indices, as well as with biochemical markers of organ dysfunction. These findings underscore the relevance of LPS as a biomarker of systemic inflammation and disease severity. Furthermore, the association between LPS levels and the presence of pathogenic bacterial taxa reinforces the central role of LPS in the pathogenesis of ACLF. These findings highlight that the regulation of inflammation, largely orchestrated by the intestinal microbiota, constitutes one of the major challenges in managing this disease.

On the other hand, predictive analysis of functional metabolic pathways in the ACLF patients indicates important adaptations of the microbiota to the intestinal microenvironment, such as increases in toluene degradation and chorismate biosynthesis. These pathways suggest a microbial response to environmental stress and are essential for the synthesis of aromatic amino acids such as L-tryptophan, L-phenylalanine, and L-tyrosine [61,62,63,64]. These metabolic pathways, which involve the synthesis of aromatic amino acids, suggest an association with stress factors originating in the intestinal microenvironment. In bacteria, these amino acids are not only essential for protein translation but also participate in the synthesis of protein factors and secondary metabolites that mediate microbial competition, interbacterial signaling and stringent response [40,65]. Likewise, the increase in bacterial pathways related to the biosynthesis of ubiquinones, key molecules in the neutralization of oxidative stress from reactive oxygen species produced by the host, could play a crucial role in the adaptation of the intestinal microbiota to the innate immune response of the host produced by the host, in response to inflammatory processes [66,67].

These findings emphasize the hypothesis that the intestinal microbiota in the context of decompensated cirrhosis and ACLF activates compensatory metabolic pathways to the host microenvironment [45], which in certain pathogens—such as *Klebsiella* and *Escherichia*—are accompanied by a stringent response, associated with increases in the expression of virulence factors and antibiotic resistance.

Some studies have described the intestinal microbiota in patients with sepsis [68,69,70]. However, to our knowledge, this is the first study to describe changes in the intestinal microbiota in patients with ACLF and sepsis. We observed a significant decrease in alpha diversity, an increase in the Proteobacteria/Firmicutes ratio, and a predominance of genera such as *Klebsiella*, *Enterococcus*, *Lactobacillus*, and *Streptococcus*. Previous reports indicate that Enterococcus and Klebsiella are the predominant genera in patients with sepsis, even in the absence of underlying pathologies [71]. This may be a consequence of bacterial translocation and the fact that these bacteria are commonly present in the hospital setting and are considered to cause nosocomial infections. In this sense, we observed that the increase in enterobactin biosynthesis in the ACLF group goes hand in hand with increases in *Escherichia/Shigella* and *Klebsiella.* This is interesting given that it has been observed that virulent and hypervirulent strains of *K. pneumoniae* are characterized by the production of this compound [72] and, remarkably, that the exacerbated production of siderophores constitutes a characteristic feature of strains of *K. pneumoniae* that cause sepsis [73].

Therefore, it is important to determine whether the bacterial colonization found in these patients is due to an in-hospital or community-acquired process, which could positively impact the rational use of antibiotics. This is especially relevant in intensive care units, where infection by multidrug-resistant bacteria in patients with decompensated cirrhosis [74], and ACLF is associated with sepsis and poor prognosis.

Sepsis is a complex syndrome with high morbidity and mortality. We validated two intestinal bacterial genera that could support the diagnosis of sepsis in ACLF. Interestingly, we found that the *Klebsiella/Faecalibacterium* ratio can be used as a biomarker for this purpose. Using ROC curves, this ratio showed an area under the curve (AUROC) of 0.83 (*p* < 0.001), which, using a cutoff >0.67, showed a sensitivity of 81.8% and a specificity of 83.3%. This ratio has not been previously described in the literature and could be a promising tool for the early detection of sepsis in patients with ACLF, a setting in which early identification is essential to reduce fatal clinical outcomes.

Another pioneering finding in the present study is the analysis of the intestinal microbiota in relation to different organ failures, specifically circulatory and liver failure, and coagulopathy. The analysis revealed a marked divergence in beta diversity, evidencing significant differences between ACLF patients with and without organ failure. The taxonomic profiles of the patients showed a predominance of *Proteus* and *Morganella* in those with coagulopathy and circulatory failure, while in patients with liver failure, taxa such as *Klebsiella*, *Prevotella*, *Fusobacterium*, and *Veillonella* were found, suggesting specific patterns of dysbiosis related to each type of organ failure. *Proteus* and *Morganella*, members of the Enterobacteriaceae family, have been commonly associated with nosocomial infections, especially urinary tract infections and bacteremia [75]. These bacteria possess hemolysins and a high capacity for cell adhesion [76], which facilitate tissue invasion and the development of serious systemic infections. On the other hand, their ability to develop antibiotic resistance [77] could increase their prevalence, as well as complicate their clinical management and increase the risk of short-term mortality. However, the association we describe of these bacterial groups with coagulopathy and circulatory failure needs future studies to determine their possible causal role.

Likewise, the role of *Prevotella* in the pathophysiology of ACLF is still unclear, but its association with dysbiosis in disease progression is well documented. In healthy individuals, *Prevotella* degrades dietary fiber, producing propionate [78]. However, in patients with cirrhosis, its abundance is significantly increased and associated with low-grade inflammation [4,14]. This pattern persists in ACLF, where *Prevotella* is statistically significantly associated with liver failure (Figure 5). However, further studies are required to understand its impact on the disease.

Furthermore, few studies have explored the correlation between intestinal microbiota and mortality in patients with cirrhosis and ACLF [6,7,79]. Most of these studies have been conducted in European and Asian populations. However, this study represents groundbreaking evidence in Mexico, specifically in western Mexico. In this regard, our results indicate that the cluster comprising *Escherichia/Shigella*, *Lactobacillus*, *Veillonella*, *Klebsiella*, and *Ruminococcus gnavus* is closely associated with 28- and 90-day mortality in patients with ACLF. This finding is partially in line with previous studies, such as that of Solé et al., who identified *Enterococcus faecium*, *Streptococcus thermophilus*, and *Ruminococcus lactaris* as predictors of low 3-month survival probability in patients with liver cirrhosis of various etiologies [7]. Likewise, Maslennikov et al. and Sung et al. reported that the Lactobacillaceae family and the *Lactobacillus* genus were associated with a higher risk of 1-year mortality in Russian and Asian populations with cirrhosis, mainly of alcoholic etiology [79,80].

Importantly, the Kaplan–Meier analysis revealed unprecedented findings in our cohort of patients with ACLF. Specifically, the presence of *Veillonella* >4.5 ASVs increases the risk of 28-day mortality by 3.16 times in ACLF patients, while *Ruminococcus gnavus* with values greater than 3.5 ASVs reduces survival to 33% at 90 days. Similarly, *Lactobacillus* with values greater than 532.5 ASVs reduces survival to only 25%, also at 90 days. These results highlight the importance of the intestinal microbiota as a potential prognostic marker in patients with ACLF and suggest the need for further studies in different geographic and demographic contexts.

On the other hand, the genus *Veillonella* comprises several pathobionts that inhabit oral cavity, whose ability to create biofilms has been well described [81]. Its pathological role in cirrhosis has been linked to an increase in intestinal dysbiosis and related complications, such as the recurrence of hepatic encephalopathy events. Furthermore, it has been proposed as a biomarker of dysbiosis in individuals undergoing prolonged treatment with proton pump inhibitors [82]. In the context of alcoholic hepatitis, *V. atypica* bacteremia has been documented, which has significant implications for mortality (HR = 2.73; 95% CI: 1.19–6.3; *p* < 0.01) [79,82,83,84,85,86]. These findings underscore the need to further explore the impact of *Veillonella* on the progression of ACLF, as well as its potential use as clinical biomarkers or therapeutic targets in the management of the disease.

Furthermore, *Ruminococcus gnavus*, whose abundance is associated with a reduction in the survival of ACLF patients, is a Gram-positive bacterium that has been associated with multiple pathologies [87]. Its mucolytic capacity and link with increased mortality in patients with fecal peritonitis secondary to small bowel perforation have been previously documented [88]. However, its association with pathologies such as ACLF has not been described, which highlights the detrimental role of certain microorganisms in contexts of intestinal dysbiosis and immunocompromise.

Finally, it is important to highlight the role of *Lactobacillus*, which has been observed to exert immunomodulatory effects in healthy subjects, reducing IL-6 production and increasing IL-10 production, contributing to intestinal homeostasis. These anti-inflammatory effects have led to the use of several species and strains of this genus as probiotics in different clinical settings [89]. Nonetheless, it is crucial to note that *Lactobacillus* presents important intrinsic resistance to certain antibiotics, such as vancomycin or some aminoglycosides, which reinforces its role as a clinically relevant pathobiont [90,91]. Some case reports have shown that in immunocompromised patients or with advanced cirrhosis, bacteria of the genus *Lactobacillus* can cause severe systemic infections, such as spontaneous bacterial peritonitis, bacteremia and endocarditis [92,93]. This highlights the need for further research in patients with ACLF, since the disruption of the intestinal barrier, severe immunocompromise, combined with the uncontrolled use of probiotics, can significantly increase the risk of *Lactobacillus*-associated systemic infections in this population.

Some of the limitations of this study include its small sample size, which may make it difficult to generalize the results to a broader population. As a cross-sectional study, it only allows for establishing associations, without determining causality or observing temporal changes in microbiota or serum LPS levels related to the presence of ACLF. Furthermore, 16S rRNA gene sequencing limits identification at the genus level, which prevents precise differentiation between bacterial species or strains, and does not allow for a complete distinction between the bacterial genera *Escherichia* and *Shigella*. Another important limitation was the lack of an in-depth dietary examination, which could have influenced the results. Nevertheless, this is a difficult aspect to assess, given the cognitive impairments that ACLF patients often present. Another important variable to consider in this study is the use of antibiotics in the majority of patients with ACLF (81.8%), compared to 18.2% without antibiotics. Although alpha and beta diversity did not differ between these two groups, the small number of subjects without antibiotics probably prevented us from identifying alterations in the intestinal microbiota in terms of the richness or dissimilarity of bacterial diversity. Antibiotics undoubtedly have negative impacts on intestinal homeostasis. Unfortunately, this variable is very difficult to assess in patients with ACLF because most patients receive antibiotics for suspected or confirmed bacterial infections and are, therefore, generally under these treatment regimens. Consequently, it is an intrinsic limitation of the present study.

## 5. Conclusions

Our results demonstrate that the intestinal microbiota of patients with ACLF is profoundly altered, based on alpha- and beta-diversity indices. This is associated with increased proliferation of bacteria from the Proteobacteria phyla, specifically *Escherichia/Shigella* and *Klebsiella*; interestingly, *Lactobacillus* is also prominent. One of the alpha-diversity metrics that assesses dominance, such as the Strong index, in conjunction with the observed taxonomic changes, showed significant associations with higher 28-day and 90-day mortalities. Therefore, alpha diversity emerges as a marker not only of dysbiosis but also of prognosis in patients with ACLF. Furthermore, we identified, for the first time in the global literature, the correlations of pathobiont, inflammatory, and opportunistic bacterial genera with sepsis, organ failure, and short-term mortality in one of the most severe cirrhosis scenarios, such as ACLF. We highlight the *Klebsiella/Faecalibacterium* ratio as a promising biomarker for the detection of sepsis, because of its high accuracy. This ratio will require further validation, such as prospective studies. The LPS levels and their different correlations reinforce the central role of bacterial translocation in the pathogenesis of ACLF, being directly associated with clinical severity and organ dysfunction. A particularly relevant finding was that more pronounced dysbiosis, especially associated with increased *Veillonella*, significantly impacted the 28-day mortality risk in ACLF, increasing it 3.03-fold. These identified intestinal microbiota-based biomarkers could complement conventional prognostic assessment algorithms, such as CLIF-SOFA, which could improve risk stratification and clinical management of patients. This study provides pioneering findings in Mexico and Latin America, laying the groundwork for future research in the region and opening new opportunities for precision medicine in advanced cirrhosis based on intestinal microbiota profiling. Future longitudinal studies will be necessary to better understand the role of the intestinal microbiota in ACLF.

## Figures and Tables

**Table 1:** Demographical and clinical characteristics of both study groups.

Characteristic	HC (*n* = 20)	ACLF (*n* = 33)	*p*-Value
Age (years)	50.65 ± 10.13	49.12 ± 10.63	0.600 ^a^
BMI (kg/m^2^)	24.9 (24.4–27.2)	23.8 (20.9–26.2)	0.200 ^b^
Hemoglobin (g/dL)	14.8 (14.0–15.6)	9.02 (7.6–11.2)	<0.001 ^b^
Platelets (10 ^9^ /µL)	231 (178.8–274.8)	92.9 (78.3–123.4)	<0.001 ^b^
White blood cells (10 ^9^ /µL)	5.9 (4.7–6.6)	13.09 (7.7–22.3)	<0.001 ^b^
Neutrophils (10 ^9^ /µL)	3.4 (2.4–4.08)	11.3 (5.7–21.7)	<0.001 ^b^
Lymphocytes (10 ^9^ /µL)	1.7 (1.5–2.1)	0.91 (0.6–1.3)	<0.001 ^b^
Total bilirubin (mg/dL)	0.6 (0.5–0.8)	8.2 (4.2–23.2)	<0.001 ^b^
Direct bilirubin (mg/dL)	0.12 (0.1–1.15)	3.5 (1.3–10.3)	<0.001 ^b^
GGT (IU/L)	23.5 (16.0–34.2)	86 (27.2–166.5)	0.003 ^b^
Albumin (g/dL)	4.5 (4.3–4.6)	2.1 (1.7–2.2)	<0.001 ^b^
ALT (IU/L)	26 (15.5–37.7)	28 (22–45)	0.180 ^b^
AST (IU/L)	20 (15.2–29.7)	68 (55–104)	<0.001 ^b^
ALP (IU/L)	73.5 (61.2–79)	163 (83–200)	<0.001 ^b^
Total protein (g/dL)	7.1 (6.8–7.6)	6 (4.8–6.4)	<0.001 ^b^
Creatinine (mg/dL)	0.78 (0.7–0.9)	2.5 (1.1–3.8)	<0.001 ^b^
Prothrombin time (s)	11.4 (11.1–12.1)	25.2 (20.2–32.3)	<0.001 ^b^
INR	1.07 (1.06–1.2)	2.3 (1.8–2.6)	<0.001 ^b^
Sodium (mmol/L)	N/A	128 (124–135)	N/A
Child–Pugh class A/B/C	N/A	0/0/33	N/A
Child–Pugh score	N/A	12 (11–13)	N/A
MELD-Na score	N/A	33.5(28–38)	N/A
ACLF Grades I/II/III	N/A	9/16/8	N/A
CLIF-SOFA	N/A	48 (39–51)	N/A
Platelet-to-neutrophil ratio	1.7(1.2–2.2)	8.9 (6.2–14.3)	<0.001 ^b^
Neutrophil-to-lymphocyte ratio	1.8 (1.5–2.2)	9.2 (6.5–21.1)	<0.001 ^b^
APRI index	0.19 (0.13–0.29)	1.5 (1–2.3)	<0.001 ^b^
FIB-4 index	0.82 (0.63–1.57)	7.4 (4.5–10.4)	<0.001 ^b^
Presence of hepatic encephalopathy (*n*, %)	N/A	28 (84.8)	N/A
West-Haven grade (1/2/3/4)	N/A	4/11/10/3	N/A
Ascites (*n*, %)	N/A	22 (66.7)	N/A
Upper gastrointestinal bleeding (*n*, %)	N/A	9 (27.2)	N/A
Acute kidney injury (*n*, %)	N/A	20 (60.6)	N/A
Lactulose (*n*, %)	N/A	31 (93.9)	N/A
Mean arterial pressure (mmHg)	N/A	75 (65–83)	N/A
Antibiotic usage (*n*, %)	N/A	27 (81.8)	N/A
Ceftriaxone (*n*, %)	N/A	21 (65.6)	N/A
Rifaximin (*n*, %)	N/A	14 (46.7)	N/A
Piperacillin/tazobactam (*n*, %)	N/A	5 (15.6)	N/A
Others (*n*, %)	N/A	5 (15.6)	N/A
Use of proton pump inhibitors (*n*, %)	N/A	22 (66.7)	N/A
Infection at admission (*n*, %)	N/A	20 (60.6)	N/A
Active alcoholism (*n*, %)	N/A	15 (45.5)	N/A
Urinary tract infection (*n*, %)	N/A	6 (18.2)	N/A
Spontaneous bacterial peritonitis (*n*, %)	N/A	6 (18.2)	N/A
Pneumonia (*n*, %)	N/A	3 (9.1)	N/A
SIRS (*n*, %)	N/A	13 (39.4)	N/A
Sepsis (*n*, %)	N/A	10 (30.3)	N/A
Septic shock (*n*, %)	N/A	4 (12.1)	N/A
Kidney failure (*n*, %)	N/A	19 (57.5)	N/A
Brain failure (*n*, %)	N/A	13 (39.3)	N/A
Liver failure (*n*, %)	N/A	14 (42.4)	N/A
Circulatory failure (*n*, %)	N/A	10 (30.3)	N/A
Respiratory failure (*n*, %)	N/A	3 (9.1)	N/A
Coagulopathy (*n*, %)	N/A	11 (33.3)	N/A
7-day mortality (*n*, %)	N/A	7 (21.2)	N/A
28-day mortality (*n*, %)	N/A	13 (39.4)	N/A
90-day mortality (*n*, %)	N/A	20 (60.6)	N/A

Abbreviations: N/A (not assessed), BMI (body mass index), GGT (gamma-glutamyl transferase), AST (aspartate aminotransferase), ALT (alanine aminotransferase), ALP (alkaline phosphatase), INR (international normalized ratio), and SIRS (systemic inflammatory response syndrome). Data are expressed as the mean ± standard deviation, median (interquartile range), or as the number of patients (%). Other antibiotics included (clarithromycin, piperacillin/tazobactam, linezolid, metronidazole, levofloxacin, and ciprofloxacin). ^a^ Student’s *t*-test. ^b^ Mann–Whitney U test. Healthy controls (HCs); acute-on-chronic liver failure (ACLF).

## Data Availability

The original contributions presented in this study are included in the article/Supplementary Material. Further inquiries can be directed to the corresponding author.

## Supplementary Materials

The following supporting information can be downloaded at: https://www.mdpi.com/article/10.3390/microorganisms13051138/s1, Figure S1: Relative abundance at the genus level; Figure S2: Beta diversity of the intestinal microbiota in patients with ACLF associated with organ failure; Table S1: Multiple comparisons of alpha-diversity values in patients with ACLF, according to the ACLF grade and other clinical variables; Table S2: Multiple comparisons of beta-diversity indices in patients with ACLF, according to the ACLF grade and other clinical variables; Table S3: Multiple comparisons of beta-diversity indices in patients with ACLF, in the presence and absence of organ failure (coagulopathy, circulatory failure, and liver failure).
