# Supplementary material for: Deciphering the Language of Intestinal Microbiota Associated with Sepsis, Organ Failure, and Mortality in Patients with Alcohol-Related Acute-on-Chronic Liver Failure (ACLF): A Pioneer Study in Latin America
Source: Microorganisms. 2025 May 15;13(5):1138. doi: 10.3390/microorganisms13051138 (PMC12113797; doi:10.3390/microorganisms13051138)
Supplement: Supplementary file 1 [file microorganisms-13-01138-s001.zip › microorganisms-3594965-supplementary.pdf]

Supplementary material

Supplementary Table S1: Multiple comparisons of alpha diversity values of intestinal microbiota in patients with ACLF, according to ACLF grade and other clinical variables. The table shows the alpha diversity values of intestinal microbiota in patients classified according to ACLF grade (I, II, and III) and the presence or absence of relevant clinical variables such as ascites, hepatic encephalopathy, AKI, infection, or antibiotic use. *p* and *q* values were calculated using the Kruskal-Wallis test with the Benjamini-Krieger-Yekutieli multiple test. acute kidney injury (AKI), Acute-on-chronic liver failure (ACLF).

	**Group 1**	**Group 2**	***H***	***p-value***	***q* -Value**
		**Observed features**			
**ACLF grades**	ACLF I	ACLFII	0.736	0.391	0.559
	ACLF I	ACLF III	0.227	0.634	0.704
	ACLF II	ACLF III	3.982	0.046	0.092
**Ascites**	Absence	Presence	3.039	0.081	0.081
**Hepatic encephalopathy**	Absence	Presence	0.709	0.400	0.400
**AKI**	Absence	Presence	1.122	0.289	0.289
**Infection**	Absence	Presence	0.679	0.410	0.410
**Antibiotic use**	Absence	Presence	0.006	0.936	0.936
		**Shannon**			
**ACLF grades**	ACLF I	ACLFII	0.445	0.505	0.683
	ACLF I	ACLF III	0.339	0.560	0.683
	ACLF II	ACLF III	1.889	0.169	0.339
**Ascites**	Absence	Presence	4.63	0.031	0.031
**Hepatic encephalopathy**	Absence	Presence	2.198	0.138	0.138
**AKI**	Absence	Presence	0.733	0.392	0.392
**Infection**	Absence	Presence	2.858	0.091	0.091
**Antibiotic use**	Absence	Presence	0.585	0.444	0.444
		**Faith phylogenetic distance**			
**ACLF grades**	ACLF I	ACLFII	1.865	0.172	0.287
	ACLF I	ACLF III	0.07	0.791	0.879
	ACLF II	ACLF III	2.04	0.153	0.287
**Ascites**	Absence	Presence	1.78	0.182	0.182
**Hepatic encephalopathy**	Absence	Presence	0.124	0.725	0.725
**AKI**	Absence	Presence	1.54	0.215	0.215
**Infection**	Absence	Presence	0.561	0.454	0.454
**Antibiotic use**	Absence	Presence	0.05	0.823	0.823
		**Strong dominance index**			
**ACLF grades**	ACLF I	ACLFII	0.445	0.505	0.721
	ACLF I	ACLF III	0.07	0.791	0.791
	ACLF II	ACLF III	0.611	0.435	0.721
**Ascites**	Absence	Presence	0.535	0.464	0.464
**Hepatic encephalopathy**	Absence	Presence	0.023	0.880	0.880
**AKI**	Absence	Presence	0.927	0.336	0.336
**Infection**	Absence	Presence	1.594	0.207	0.207
**Antibiotic use**	Absence	Presence	0.102	0.750	0.750

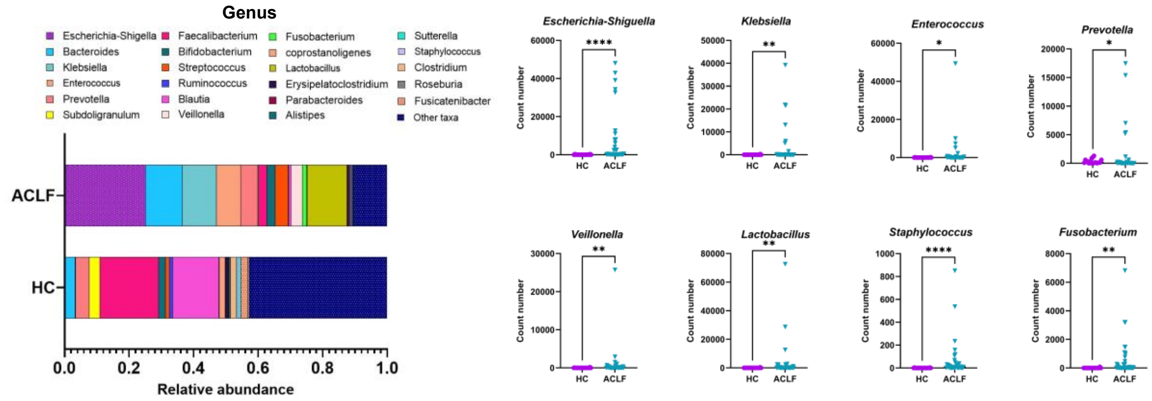

Supplementary Figure S1: (a) Relative abundances of intestinal microbiota at the genus level, (b) Scatter plots showing the differences between the numbers of ASVs from representative bacterial genera such as *Escherichia*/*Shigella*, *Klebsiella*, *Enterococcus*, *Prevotella*, *Veillonella*, *Lactobacillus*, *Staphylococcus*, and *Fusobacterium* in the healthy control (HC, represented in purple) and ACLF patients (represented in blue) groups. ASV counts were previously transformed using centered log-ratio. Statistical analyses were performed using the Mann–Whitney U test. Significance levels are indicated as $p < 0.05$ (*), $p < 0.01$ (**), $p < 0.001$ (***), and $p < 0.0001$ (****). Healthy controls (HC), Acute-on-chronic liver failure (ACLF).

Supplementary Table S2: Multiple comparisons of intestinal microbiota beta diversity indices in patients with ACLF, according to ACLF grade and other clinical variables. Comparisons and statistical distances (pseudo-F) were calculated and compared using PERMANOVA. Multiple comparison analyses (*q*) were performed using Benjamini-Hochberg test. Acute kidney injury (AKI), Acute-on-chronic liver failure (ACLF).

		**Unweighted Unifrac**			
	**Group 1**	**Group 2**	**Pseudo-F**	***p-value***	***q-value***
**ACLF grades**	ACLF I	ACLFII	1.264	0.203	0.290
	ACLF I	ACLF III	0.957	0.447	0.447
	ACLF II	ACLF III	1.646	0.096	0.192
**Ascites**	Absence	Presence	1.461	0.137	0.137
**Hepatic encephalopathy**	Absence	Presence	1.227	0.231	0.231
**AKI**	Absence	Presence	1.09	0.281	0.281
**Infection**	Absence	Presence	0.755	0.662	0.662
**Antibiotic use**	Absence	Presence	0.545	0.947	0.947
		**Weighted Unifrac**			
	**Group 1**	**Group 2**	**Pseudo-F**	***p-value***	***q-value***
**ACLF grades**	ACLF I	ACLFII	1.354	0.228	0.380
	ACLF I	ACLF III	0.250	0.984	0.989
	ACLF II	ACLF III	1.136	0.316	0.451
**Ascites**	Absence	Presence	0.715	0.603	0.603
**Hepatic encephalopathy**	Absence	Presence	1.674	0.118	0.118
**AKI**	Absence	Presence	1.629	0.147	0.147
**Infection**	Absence	Presence	2.5	0.039	0.039
**Antibiotic use**	Absence	Presence	0.545	0.947	0.947
		**Bray-Curtis**			
	**Group 1**	**Group 2**	**Pseudo-F**	***p-value***	***q-value***
**ACLF grades**	ACLF I	ACLFII	1.453	0.086	0.172
	ACLF I	ACLF III	1.143	0.309	0.515
	ACLF II	ACLF III	0.742	0.843	0.940
Ascites	Absence	Presence	1.561	0.049	0.049
**Hepatic encephalopathy**	Absence	Presence	1.266	0.156	0.156
**AKI**	Absence	Presence	0.768	0.809	0.809
**Infection**	Absence	Presence	1.049	0.331	0.331
**Antibiotic use**	Absence	Presence	0.780	0.844	0.844
		**Jaccard**			
	**Group 1**	**Group 2**	**Pseudo-F**	***p-value***	***q-value***
**ACLF grades**	ACLF I	ACLFII	0.906	0.849	0.943
	ACLF I	ACLF III	1.052	0.235	0.470
	ACLF II	ACLF III	1.007	0.407	0.642
**Ascites**	Absence	Presence	1.118	0.121	0.121
**Hepatic encephalopathy**	Absence	Presence	1.091	0.159	0.159
**AKI**	Absence	Presence	1.015	0.396	0.396
**Infection**	Absence	Presence	0.915	0.820	0.820
**Antibiotic use**	Absence	Presence	0.941	0.732	0.732

Analysis of β diversity associated with organ failure.

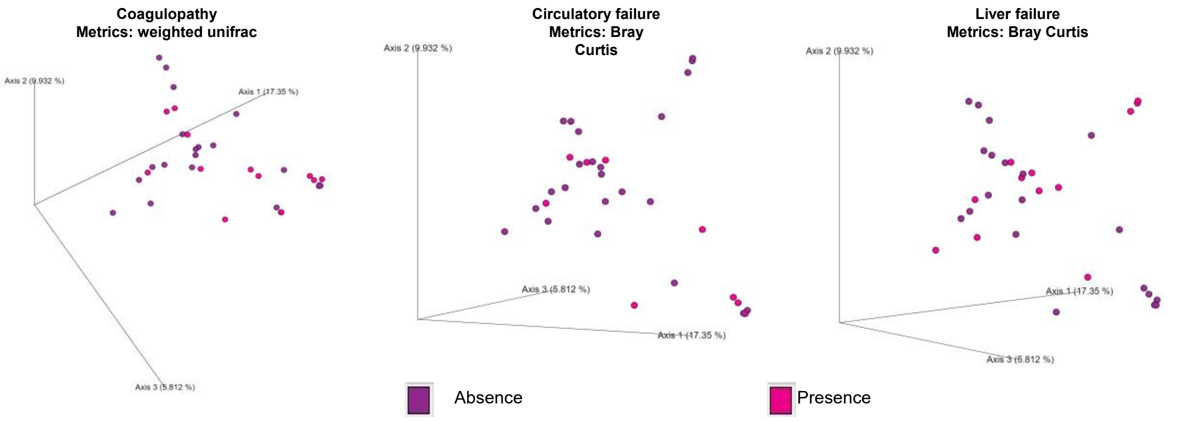

Supplementary Figure S2: Beta diversity of intestinal microbiota in patients with ACLF associated with organ failure. Beta diversity analysis in ACLF, in the presence and absence of organ failure (coagulopathy, circulatory failure, and liver failure). A three-dimensional scatter plot is shown using PCoA. ACLF patients with fault (pink dots) and ACLF patients without fault (purple dots). Analyzed with PERMANOVA, $q < 0.001$ in both cases. Acute-on-chronic liver failure (ACLF).

Supplemental table S3: Multiple comparisons of intestinal microbiota beta diversity indices in patients with ACLF, in the presence and absence of organ failure (coagulopathy, circulatory failure, liver failure, kidney failure, brain failure and respiratory failure). Statistical analyses and calculation of *p*-values were performed using the PERMANOVA test with Benjamini-Hochberg (BH) multiple testing correction (*q*-values).

	**Unweighted Unifrac**		
	**Pseudo-F**	***p*-value**	***q*-value**
Kidney failure	0.731	0.709	0.709
Brain failure	0.836	0.534	0.534
Respiratory failure	0.974	0.451	0.451
Circulatory failure	0.721	0.758	0.758
Coagulopathy	0.994	0.393	0.393
Liver failure	1.281	0.174	0.174
	**Weighted Unifrac**		
	**Pseudo-F**	***p*-value**	***q*-value**
Kidney failure	1.423	0.216	0.216
Brain failure	0.224	0.986	0.986
Respiratory failure	0.953	0.399	0.399
Circulatory failure	0.721	0.758	0.750
Coagulopathy	2.03	0.054	0.054
Liver failure	0.559	0.743	0.743
	**Bray-Curtis**		
	**Pseudo-F**	***p*-value**	***q*-value**
Kidney failure	0.802	0.743	0.743
Brain failure	0.706	0.864	0.864
Respiratory failure	1.231	0.157	0.157
Circulatory failure	1.496	**0.053**	**0.053**
Coagulopathy	0.926	0.500	0.500
Liver failure	1.681	**0.039**	**0.039**
	**Jaccard**		
	**Pseudo-F**	***p*-value**	***q*-value**
Kidney failure	1.007	0.410	0.410
Brain failure	0.974	0.561	0.561
Respiratory failure	1.034	0.322	0.322
Circulatory failure	0.934	0.754	0.754
Coagulopathy	0.970	0.588	0.588
Liver failure	1.014	0.384	0.384
